# Development of Antimicrobial Packaging Film Made from Poly(Lactic Acid) Incorporating Titanium Dioxide and Silver Nanoparticles

**DOI:** 10.3390/molecules22071170

**Published:** 2017-07-13

**Authors:** Wenhui Li, Cheng Zhang, Hai Chi, Lin Li, Tianqing Lan, Peng Han, Haiyan Chen, Yuyue Qin

**Affiliations:** 1 Institute of Yunnan Food Safety, Kunming University of Science and Technology, Kunming 650550, China; 15559823733@163.com (W.L.); 13136640259@163.com (C.Z.); 18468273140@163.com (H.C.); lantianqing_8019@163.com (T.L.); hanpeng320@163.com (P.H.); seacome@163.com (H.C.); 2 College of Light Industry and Food Science, South China University of Technology, Guangzhou 510640, China; felinli@scut.edu.cn

**Keywords:** polylactide (PLA), nano-TiO_2_, nano-Ag, antimicrobial activity, migration properties

## Abstract

Polylactide (PLA)/nano-TiO_2_ and PLA/nano-TiO_2_/nano-Ag blends films were prepared by a solvent volatilization method. Compared to pure PLA film, the nano-blend films have low water vapor permeability (WVP) and a poor transparency. With the increase of the NPs in the PLA, the tensile strength (TS) and elastic modulus (EM) decreased, while the elongation at break (ε) increased. SEM analysis indicated a rougher cross-section of the nano-blend films. According to the FTIR analysis, no new chemical bonds were formed in the nano-blend films. By using DSC to examine the crystallization and melting behavior, the result shows that the NPs have no effect on the glass transition (T_g_) and melting temperature (T_m_), but they caused an increase on the cold crystallization (T_c)_ and crystallinity (*X_c_*). TGA results show that the addition of nanoparticles significantly improved the thermal stability. The PLA nano-blend films show a good antimicrobial activity against. *E. coli* and *Listeria monocytogenes*. Most important, we carried out migration tests, and verified that the release of NPs from the nano-blend films was within the standard limits.

## 1. Introduction

In last decades, a large number of plastic packaging bags have been produced from fossil fuels, and environmental pollution has become a global issue. One important way to solve the problem is to produce packaging bags using degradable materials [1]. Because the use of bio-based antimicrobial packaging materials can reduce the environment impacts, and offer protection for the produce against physical, chemical, and microbiological effects, bio-based materials have attracted extensive interest in the packaging field [2].

Among the various biopolymers, poly(lactic acid) (PLA) which can be produced from the bacterial fermentation of normal renewable resources, like corn starch or sugar beet, and can be eventually decompose when it is buried is the ideal choice to achieve these goals [3]. Furthermore, due to its high modulus, high strength, thermoplasticity, and biodegradability, the polymer is widely been used as a packaging material [4]. Because PLA has been approved by the United States Food and Drug Administration (FDA) as well as the current European legislation for the use in food-contact materials, it can be used to package food. However, pure PLA materials also have some shortcomings, such as poor hydrophilicity, poor mechanical properties and lack of functionality, and these shortcomings limit their range of application. In order to make PLA a lot more competitive compared to other commodity plastic films, a lot of work has been done to improve its properties. One of the most important targets is to add functional agents, such as tea polyphenols, chitosan and other substances, which can improve the antibacterial properties of polylactic acid nanofiber membranes [5]. Some suitable nanoparticles (NPs) incorporated within the PLA matrix also can impart new functionalities to PLA-based antimicrobial packaging materials [6].

Titanium dioxide plays an important role in various modern industry and application fields. Apart from its chief use as an excellent white inorganic pigment in paint production, titanium dioxide is an important compound in the food industry, pharmacy, cosmetics, textile industry, ceramics and other modern technologies [7]. Nano-TiO_2_ has good photocatalytic activity, broad-spectrum antibacterial and UV protection functions. In the field of food packaging, the photocatalytic activity of the nano-TiO_2_ is mainly used. It has been reported that by adding nano-TiO_2_ particles to high density polyethylene and other materials one could obtain useful novel materials for packing applications [8,9]. Nano-Ag particles are also a promising antimicrobial material, because Ag nanoparticles can penetrate bacteria and kill them by attaching to the cell membranes [10]. Moreover, the large surface area of Ag NPs also has an effective inhibitory effect on the growth of the bacteria, thus, they have been widely used in the textiles, water filtration, health care and food packaging fields [11].

Incorporating NPs into PLA can afford a nanocomposite material, which has good biodegradability that can effectively solve the waste disposal problem. The mechanical properties and the crystalline properties of the nanocomposites materials are obviously improved in comparison with pure PLA materials [12]. The major risk of consumer exposure to NPs from food packaging is likely to be through potential migration of NPs into food, as found for other heavy metals [13,14]. In parallel to the fast technical development in the area of consumer products, an increase in data and knowledge about interactions between nanomaterials and humans via migration from food contact materials is needed [15]. Until now, there is no standard or generally recognized methodology available for the sufficiently sensitive and unambiguous detection of NPs that migrates out of food contact materials (FCMs), and little data about the migration of NPs from FCMs to food has been published to date [15].

In this paper, the preparation of the PLA nano-composite films with different percentages of NPs, and the evaluation of the effects of these nanoparticles on the morphological, thermal, mechanical, water vapor barrier, and antimicrobial properties of PLA material are presented. More importantly, we investigated the migration of NPs from PLA nanocomposite packaging into food simulants at a given temperature and different times.

## 2. Materials and Methods

### 2.1. Materials

PLA (Mw = 280 kDa, Mw/Mn = 1.98) used in this work was purchased from Nature Works LLC (Blair, NE, USA). TiO_2_ nanoparticle powder with an average particle size <100 nm and purity of 99.5% was obtained from Sigma (St. Louis, MO, USA). Nitric acid (HNO_3_, guaranteed reagent), acetic acid (analytical reagent) and absolute ethanol (analytical reagent) were obtained from Tianjin Fengchuan Chemical Co., Ltd. (Tianjin, China). Dichloromethane was obtained from Chengdu Kelong Chemical Co., Ltd. (Chengdu, China). A standard solution of Ag and Ti (1000 mg/mL) was obtained from the National Analysis Center for Iron and Steel of China (Beijing, China).

### 2.2. Preparation of Films

All the films used in this work were prepared by the solvent volatilization method, according to our previous work [16]. Briefly, PLA resin (2 g) was dissolved in dichloromethane (50 mL). Then, a certain percentage of nanoparticles was added into the PLA dichloromethane solution and stirred for 12 h using a batch mixer. Eventually, the solution was cast onto a glass plate and dried at ambient temperature to form a film. The PLA film with 1 wt % nano-TiO_2_ was named PLA/Ti1%, and that with 5 wt % nano-TiO_2_ was named PLA/Ti5%. The PLA film with 1 wt % nano-TiO_2_ and 0.5 wt % nano-Ag were named PLA/Ti1%/Ag, and that with 5 wt % nano-TiO_2_ and 0.5 wt % nano-Ag was named PLA/Ti5%/Ag. Pure PLA film was used as control.

### 2.3. Water Vapor Permeability

The water vapor permeability (WVP) of films was examined by the ASTM E96-95 standard method [17]. Films were placed on the top of the measuring cups which contain desiccant. Then, the measuring cups were put into a desiccator, and the desiccator was placed inside a cabinet with constant temperature and humidity, which were 20 °C and 50% relative humidity (RH). The weight loss of each bottle was measured every 1 h for 24 h. For each film sample, five replications were tested, and the mean value was taken. The *WVP* of the film was calculated by the following equation [18]:

(1) $$\mathrm{WVP}\;=\frac{W\;\times \;X}{A\;\times \;T\;\times \;\Delta P}$$

where *W* is the weight gain (g); *Χ* is the film thickness (m); *A* is the area of exposed film (m^2^); *T* is time of gain (s); and ΔP is the vapor pressure differential across the film (Pa). This entire procedure was repeated thrice for each film type.

### 2.4. Mechanical Properties

Mechanical properties of the films were tested using CMT 4104 tensile testing equipment (MTS Systems Co., Ltd., Shanghai, China). All the samples were cut into 25 mm × 100 mm pieces, and the tensile speed was 50 mm/min at room temperature according to ASTM D638. An average of six test values was taken for each sample.

### 2.5. Color

The surface color of the film was detected by measuring L* (light/dark), a* (red/green), and b* (yellow/blue) using a colorimeter (WSC-S; Shanghai Precision Instrument Co., Ltd., Shanghai, China). The opacity of each film was detected by a method similar to the one used our previous study [16]. All measurements were performed in triplicate.

### 2.6. Film Microstructure

The cross-section morphology of all the films was observed by scanning electron microscopy (SEM) under high vacuum with a S-3400N SEM instrument (Hitachi Ltd., Tokyo, Japan). The method was similar to the previous work of Liu et al. [19]. Before the observation, the films were submerged in liquid nitrogen and broken, and then the films need to be coated with a 20 nm thin conductive gold layer.

### 2.7. FTIR Analysis

Before the measurement, all the films were dried in a desiccator for at least 2 weeks. Fourier Transform Infrared (FTIR) measurements were carried out by using a Nicolet-5700 FTIR spectrometer (Ametek, Inc., Shanghai, China) with a spectral resolution of 4 cm^−1^. FTIR spectra were collected from 400 cm^−1^ to 4000 cm^−1^.

### 2.8. Differential Scanning Calorimetry (DSC)

The thermal behavior of all the films were evaluated by a DSC 214 instrument (Netzsch, Selb, Germany) under an inert nitrogen stream. About 10 mg of specimen was sealed in an aluminum pan and the DSC scans recorded while heating from 10 °C to 200 °C at a heating rate of 10 °C/min, and then cooled to 10 °C. In order to eliminate the prior thermal history of the sample, we obtained the thermal properties such as the glass transition temperature (T_g_), melting temperature (T_m_) and cold crystallization temperature (T_c_) from the second heating scan. In addition, the percentage of crystallinity (*X_c_*)) was calculated according to the following Equation (2) [20]:

*X_c_* (%) = (Δ*Hm*/*W*Δ*Hm*) × 100 (2)

where Δ*Hm* (J/g) is the heat of fusion of the sample. *W*Δ*Hm* is the heat of fusion for completely crystalline PLA (93.7 J/g), and w is the weight fraction of PLA in the samples [21].

### 2.9. Thermogravimetric Analysis (TGA)

The thermal stability analysis tests of the films were carried out by using a Net-Zach DSC-200PC analyzer (Netzsch). The samples were sealed in a small ceramic cup and heated from 20 °C to 600 °C at the speed of 10 °C/min in a nitrogen environment. Weight loss of samples was measured as a function of temperature [16,19].

### 2.10. Antimicrobial Activity

We used the liquid culture test to evaluate the antimicrobial activity of the PLA/NPs film. The *Escherichia coli* and *Listeria monocytogenes* bacteria were obtained from the Faculty of Life Science and Technology, Kun Ming University of Science and Technology (Kunming, China). Firstly, the bacteria was activated by inoculation onto Mueller-Hinton broth. Each sample (0.18–0.20 g) was put into a glass test tube containing 10 mL of broth, and 0.1 mL of inoculum was inoculated into the medium in the test tube. Then, we adjusted the bacterial culture concentration to 105 CFU/mL [22]. After 12 h of constant temperature incubation, the bacterial suspensions were serially diluted with sterile phosphate buffer and then inoculated onto the Petri dishes. Finally, all the Petri dishes were incubated at 37 °C for 12 h. The bacterial growth and colony-forming units (CFU) during the incubated time were counted to measure the antimicrobial activity of each sample.

### 2.11. Migration Test

#### 2.11.1. Food Simulants

According to Council European Directive 85/572/EEC, promulgated by the European Commission in 1985, laying down the list of food simulants that can be used to test migration of constituents of plastic materials [23] 95% (*v*/*v*) aqueous ethanol and 3% (*w*/*v*) aqueous acetic acid were selected as food simulants. Briefly, all the films were cut into pieces of 4 cm × 4 cm and weighed. Then, the film sample was immersed in 30 mL of simulant solution, and kept at 25 °C for 0, 10, 20, 30, 40 days.

#### 2.11.2. ICP-AES Analysis

The determination of the titanium contents of the two simulants was performed as follows: after removing the film samples from the vials, the solution was poured into another conical flask. Then we placed the conical flask on an electric heating plate until the migrated simulant was nearly evaporated to dryness (2–3 mL), then it was diluted with 5% HNO_3_ to 10 mL. The amount of TiO_2_ and Ag NPs in the food simulant was analyzed by inductively coupled plasma atomic emission spectrophotometry (ICP-AES, Optima 8000, Perkin Elmer, Waltham, MA, USA). The amount of migration was calculated as the ratio between the amount of the NPs found in the food simulant (M) and the initial amount of nanoparticle in the PLA/nano-blend films (m). The migration analysis was carried out three times, and the result was expressed as ug/kg [24].

### 2.12. Statistical Analysis

The means of the results were analyzed by the analysis of variance (ANOVA) using the SPSS software package (version 13.0, SPSS Inc., Chicago, IL, USA). Duncan’s multiple range test was used to compare differences (*p* < 0.05).

## 3. Results and Discussion

### 3.1. WVP

**Figure 1:**
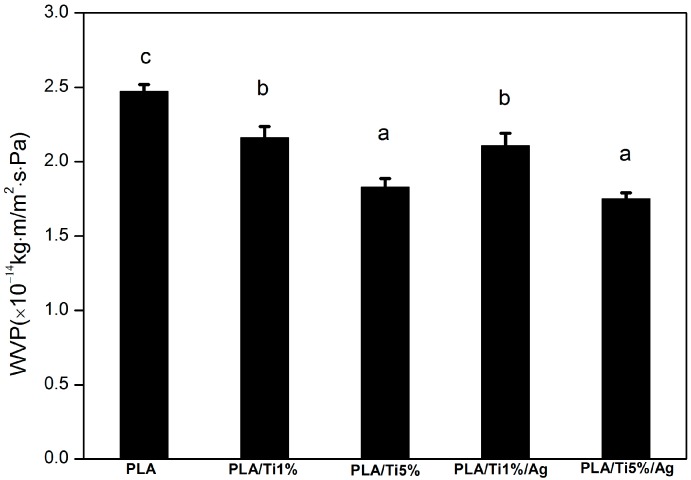
WVP analysis of pure PLA and PLA nano-blend films. Values followed by different superscript letters (a–c) in the same column were significantly different (*p* < 0.05), where a is the lowest value.

One of the most important properties of bio-based films for food packaging applications is to minimize moisture transfer from the environment to food products [25]. For this purpose, the WVP of food packaging materials should be as low as possible [26]. The WVP of the prepared films is shown in Figure 1. The WVP of PLA film was 2.47 × 10^−14^ kg·m/m^2^·s·Pa, and it was significantly (*p* < 0.05) higher than that of other films, which were 2.16 × 10^−14^ (PLA/Ti1%), 1.83 × 10^−14^ (PLA/Ti5%), 2.11 × 10^−14^ (PLA/Ti1%/Ag), and 1.75 × 10^−14^ kg·m/m^2^·s·Pa (PLA/Ti5%/Ag), respectively. The WVP of the PLA nano-blend films decreased with the increase of nanoparticles content. The low particle diameter of nanoparticles would lead to a more tortuous pathway reducing the diffusioncoefficient [27,28]. The tortuous pathway prolongs the water vapor molecule pathway and it is the main reason for the improvement of water resistance in the nano-blend films [29]. Similar results were obtained by Lizundia et al. [30]. In addition, there was no significant (*p* > 0.05) difference in WVP value when 0.5 wt % nano-Ag was added into the PLA/nano-Ti film. This behavior might be due to the hydrophilicity of nano-Ag which improved the hydrophilic interaction of films [31].

### 3.2. Mechanical Properties

When the nano-blend film is applied to preserve food, it needs to maintain its integrity and withstand external stress, so the mechanical properties are very important characteristics for the film [32]. The mechanical properties such as tensile strength (TS), elastic modulus (EM) and elongation at break (ε) of the films are listed in Table 1. The pure PLA film exhibited a higher tensile strength (TS = 15.16 MPa) and a lower percentage elongation at break (ε = 40.36%) and elastic modulus (EM = 3027.53 MPa) values compared to other films. This change led to an enhancement of the plastic elongation and reduction in brittleness compared to pure PLA film. The results were similar to those reported by Li et al. and Chrissafis et al. [33,34]. They studied PE material incorporated with the nanoparticles, and obtained similar mechanical property results. The simultaneous increase of E value and EM value of the PLA after being mixed with the nano-TiO_2_ particle may be because their sizes were closer to those of the polymer chain and the NPs acted as a crosslink between the two chains. However, the NPs added into the PLA can form a lot of cracks or cavities, which decrease the TS of the nano-blend films [35]. As presented in Table 1, with the increase of the percent of the nanoparticles from 1% to 5%, the EM and the E value of the blend film increased significantly (*p* < 0.05) and the TS value decreased slightly. A similar trend was also observed by Panaitescu et al., who studied film blends of PE mixed with nano-TiO_2_ [36]. The PLA incorporated with nano-TiO_2_ resulted in an increase of the elastic modulus of PLA due to the assignment of the high stiffness of the fillers compared to the PLA. There was no significant difference between the PLA/Ti film and PLA/Ti/Ag film in this study. This also showed that the appropriate amount of NPs in the blends led to obvious improvements in the mechanical properties of the blends, but additional increases in the amount of nano-TiO_2_ would decrease the tensile strength of PLA nano-blend films.

### 3.3. Color

The colors of all the five films are shown in Table 2. The L*, a*, b* values of the pure PLA were 52.53, −1.46 and −0.73, respectively. The L*(lightness) value of the nano-blend films was significantly (*p* < 0.05) higher than the pure PLA film. This behavior might be attributed to the white color of the nano-TiO_2_ powder. However, the L*(lightness) value of PLA/Ti nano-blend films was significantly (*p* < 0.05) higher than the PLA/Ti/Ag film, which can be explained by the argenteous sheen of the nano-Ag powder. Rhim et al. and Zeng et al. also reported that the dark color of the film will develop when the nano-Ag particle was added into the composite film [37,38].

**Figure 2:**
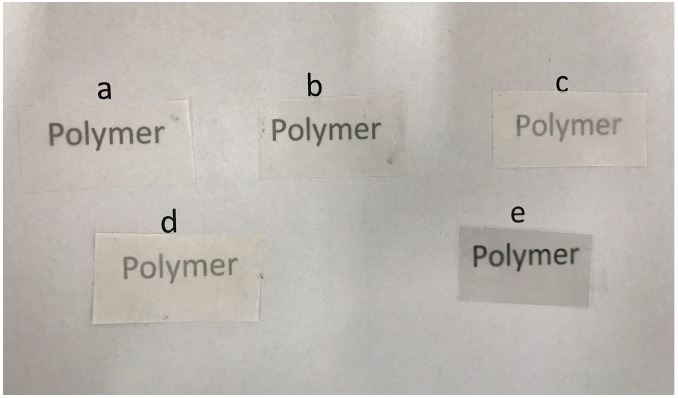
The visual properties of: (**a**) PLA/Ti1%, (**b**) PLA/Ti5%, (**c**) PLA/Ti1%/Ag, (**d**) PLA/Ti5%/Ag, and (**e**) PLA.

The opacity data of all the films are shown in Table 2, and the visual properties of the films are shown in Figure 2. The transparency of the PLA/nanoparticle films decreased with the addition of nanoparticles. This was probably due to the color of the nano-TiO_2_ and nano-Ag powder. The increase in film opacity as a consequence of the addition of nanoparticles had been reported with polymer films [39]. However, the difference in opacity among film samples was not perceptible to the human eyes. The PLA nano-blend films still had good transparency, even at high nanoparticles content. The results suggested a high transparency of PLA nano-blend films and the possibility to see through the packaging film, which is one of the most important requirements for consumers.

### 3.4. Film Microstructure

**Figure 3:**
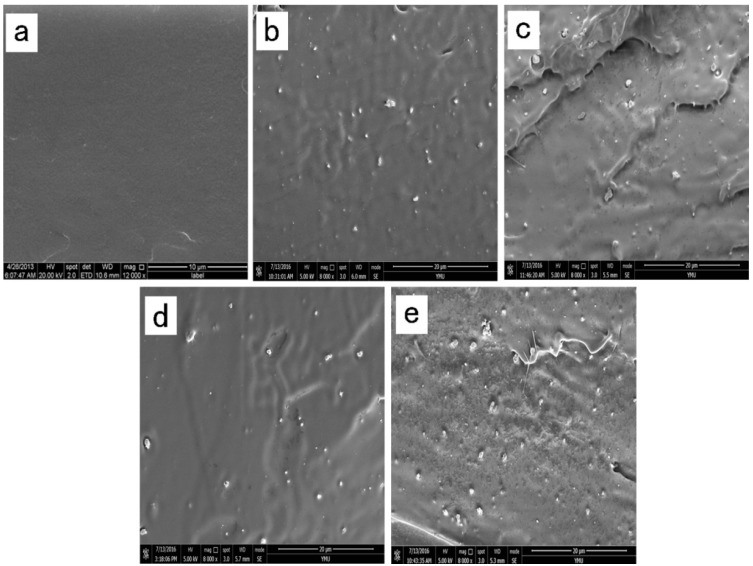
SEM micrographs of the fracture morphology of: (**a**) PLA, (**b**) PLA/Ti1%, (**c**) PLA/Ti5%, (**d**) PLA/Ti1%/Ag, and (**e**) PLA/Ti5%/Ag.

In order to investigate the dispersion and distribution of TiO_2_ NPs in the films, SEM analysis was performed. The cross-section morphology of all the films is shown in Figure 3. The representative SEM images of the pure PLA film showed a good compact structure, with a smooth and flat appearance. This phenomenon might be related with the brittleness property of pure PLA [16]. The cross-section of PLA/nanoparticle blend films (Figure 3b–e) showed rougher surfaces, and it was clearly observed that the NPs were almost completely dispersed in the PLA. On the whole, the NPs showed good dispersion in PLA at low loading levels. However, when the percent of nano-TiO_2_ increased, there had a tendency of aggregation of the nano-TiO_2_ in the material. This behavior can be explained by the interaction between the Ti–OH groups on the particle surfaces [39].

### 3.5. FTIR

**Figure 4:**
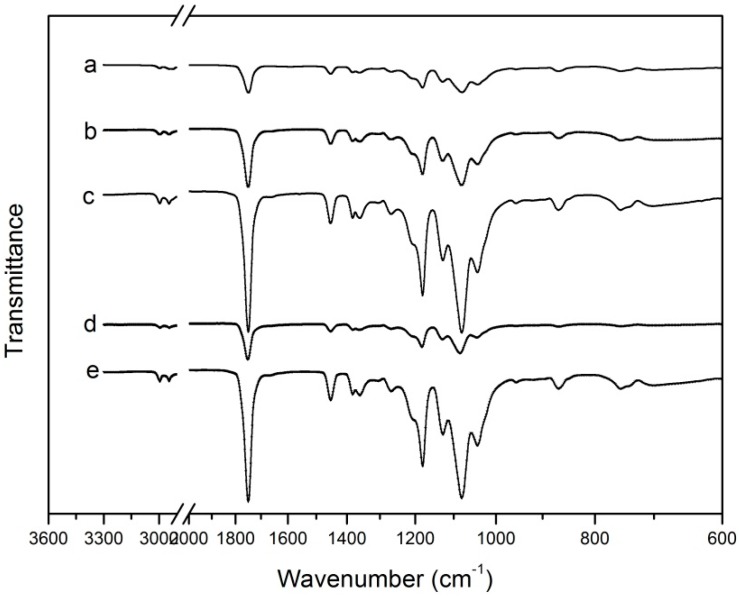
The FTIR spectra of: (a) PLA, (b) PLA/Ti1%, (c) PLA/Ti5%, (d) PLA/Ti1%/Ag, and (e) PLA/Ti5%/Ag.

FTIR spectroscopy was used to study the changes in the characteristic signals of PLA/NPs blends [16]. The infrared spectra of the PLA incorporated with different percent of NPs are given in Figure 4. The FTIR spectrum of PLA showed the following main absorption bands: the bands at 2994.5 cm^−1^ and 2977.9 cm^−1^ correspond to C–H asymmetric and symmetric stretching vibrations, respectively. The peak obtained at 1746.7 cm^−1^ (C=H), 1452.3 cm^−1^ (–CH_3_ asymmetric bending), 1382 cm^−1^ and 1359.7 cm^−1^ (–CH– deformation symmetric and asymmetric bend), 1080.4 cm^−1^ and 1180.1 cm^−1^ (C–O–C asymmetric and symmetric), 1041.5 cm^−1^ (–OH bend), 956.2 cm^−1^ (–CH3 rocking modes) and 867.1 cm^−1^ –C–C– stretch). These peaks are the typical absorption bands of PLA, and have been reported by many researchers [40].

It is easy to find from Figure 4a–e, that the strong absorption peaks of the PLA/nano-blend film show no obvious differences from those of pure PLA. Thus, we can know that the incorporation of the PLA with nano-TiO_2_ and nano-Ag particles does not result in the formation of new chemical bonds, because the addition of nano-TiO_2_ and nano-Ag do not alter the molecular and inter-molecular interaction arrangements in the polymer matrix. A similar phenomenon has been reported by Zhang et al. [41]. However, it is not difficult to observe from the Figure 4a–e, that with the increase of the NPs, the band intensity of the nano-blend film increased significantly. The behavior can be explained since the nucleation rate and nucleation density were higher than the pure PLA as the TiO_2_ and Ag NPs were added into the PLA film [39].

### 3.6. Differential Scanning Calorimetry (DSC)

**Figure 5:**
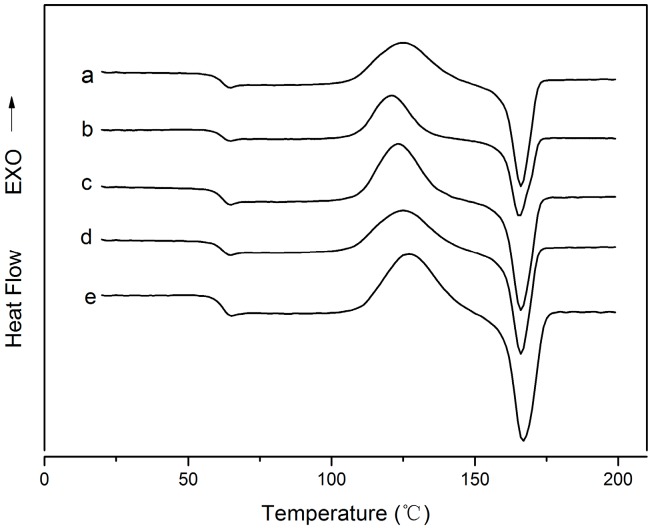
DSC curves of: (a) PLA, (b) PLA/Ti1%, (c) PLA/Ti5%, (d) PLA/Ti1%/Ag, and (e) PLA/Ti5%/Ag.

The typical differential scanning calorimetry (DSC) curves of all the five films are shown in Figure 5. The glass transition (T_g_), cold crystallization peak (T_c_) and the melting process (T_m_) can be seen in the figure and Table 3 summarizes the main information obtained from the DSC curves. It was easy to find that the nano-TiO_2_ and nano-Ag particle filler had a limited effect on the thermal transitions of the PLA. Compared to the neat PLA film, the addition of the nano-TiO_2_ and nano-Ag particles did not affect the T_g_ (58 °C) and T_m_ (160 °C) of the PLA.

This might be because the addition of the NPs did not change the mobility of the PLA macromolecular chains [42]. Luo et al. and Zhang et al. also found that the addition of nano-TiO_2_ in PLA did not result in obvious changes in the thermal transitions of PLA [42,43]. Yang et al. also reported that incorporating nano-SiO_2_ into PLA did not result in a noticeable change in T_g_ [44]. On the other hand, we can see from Figure 5 that the cold crystallization temperature (T_c_) was changed by the introduction of NPs and the introduction of the nano-TiO_2_ in PLA led to an obvious decrease in the cold crystallization temperature (T_c_). However, with the increase of the NPs, the T_c_ showed a slight recovery. This behavior was different with the finding of Luo et al., who saw no obvious change of the T_c_ when the nano-TiO_2_ loading was increased to 8 wt % [43]. It is also different from the findings of Zhang et al., who found that the higher the TiO_2_ filler load, the lower the cold crystallization temperature (T_c_) [42]. Contrarily, it is not difficult to see that with the addition of nano-Ag in the PLA/nano-TiO_2_ blends, these transition temperatures are almost unchanged. This behavior is in agreement with the studies of Lonjon et al. and Doganay et al., who all found that the addition of nano-Ag did not change the thermal transitions of the polymer [40,45]. This behavior might because the Ag nanoparticles were not agglomerated within the matrix. As listed in Table 3, the addition of the TiO_2_ nanoparticles led to a significant increase of the degree of crystallinity (*X_c_*). Moreover, the *X_c_* of the PLA/TiO_2_ films increased with the increase of nano-TiO_2_ content. This result can be explained by the phenomenon of heterogeneous nucleation. A similar result has been obtained by Buzarovska et al. and Luo et al. [43,46].

### 3.7. Thermogravimetric Analysis (TGA)

**Figure 6:**
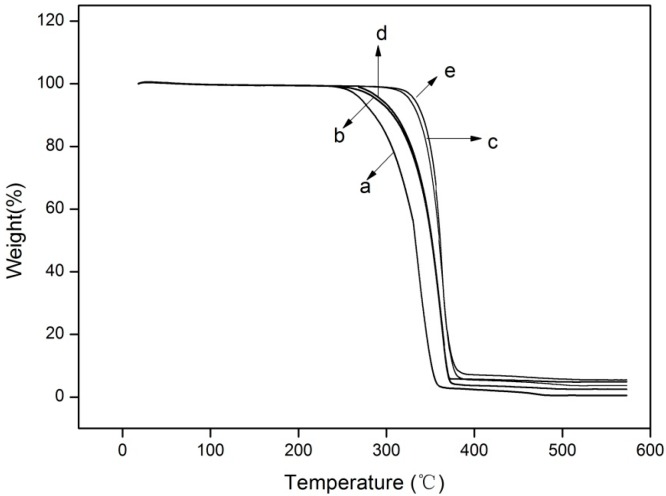
TGA curves of the PLA/NPs blend films: (a) PLA, (b) PLA/Ti1%, (c) PLA/Ti5%, (d) PLA/Ti1%/Ag, and (e) PLA/Ti5%/Ag.

TGA analysis was carried out to study the thermal stability of the films. The TGA curves of all five films are shown in Figure 6. From the thermogravimetric curves, it can be concluded that PLA and the nano-blend films all presented a relatively good thermal stability, since all the maximum mass losses occurred near 300 °C. The onset degradation temperature (T_onset_) of pure PLA was approximately 286 °C and the degradation was complete at about 360 °C. It was significantly (*p* < 0.05) lower than those of other four films. Both T_onset_ and completed degradation temperature of the other four nano-blend films were shifted to a higher value compared with the pure PLA film. In addition, the temperatures at which the degradation process was at 50% (T_50%_) of the samples were 332.75 °C, 351.74 °C, 351.18 °C, 359.80 °C and 361.21 °C for PLA, PLA/Ti1%, PLA/Ti1%/Ag, PLA/Ti5% and PLA/Ti5%/Ag, respectively. Therefore, we can conclude that the addition of the NPs obviously improved the thermal stability of the PLA film. Similar data has been reported by Zhang et al., who studied the nanocomposite film obtained by adding nano-TiO_2_ to poly(lactic acid)/sesbania gum [39]. One of the possible reasons for this behavior is that TiO_2_ NPs may act as a heat barrier in the early stages of thermal decomposition [46,47]. At 580 °C, the residue of the pure PLA film was about 0.48%, that is significantly (*p* < 0.05) lower than that of the other four films, which were 2.4%, 3.6%, 4.9% and 5.4% for PLA/Ti1%, PLA/Ti1%/Ag, PLA/Ti5% and PLA/Ti5%/Ag, respectively. The differences were in agreementwith the amount of nano-TiO_2_ added into the PLA film. Similar results have been obtained by Zhang et al. and Fei et al. [39,47].

### 3.8. Antimicrobial Activity

Antimicrobial activity of the films was evaluated by their inhibition ability against microorganism growth. Because *Escherichia coli* and *Listeria monocytogenes* are typical spoilage organism groups in food products, the inhibition ability against these two bacterial species was tested to show the antimicrobial activity of the films [19,22].

**Figure 7:**
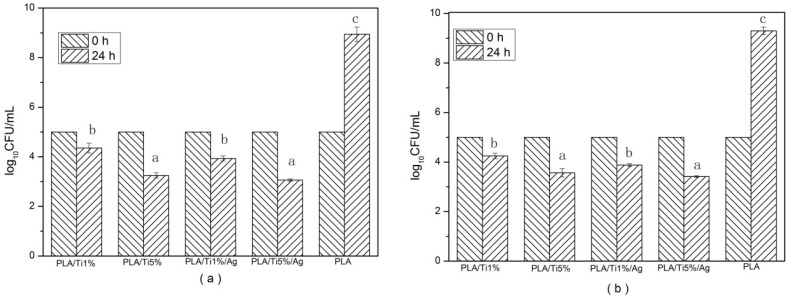
Antimicrobial activity of pure PLA and PLA nano-blend films. (**a**) *E. coli* and (**b**) *Listeria monocytogenes*. Values followed by different superscript letters (a–c) in the same column were significantly different (*p* < 0.05), where a is the lowest value.

The antimicrobial activity test results are shown in Figure 7. According to the obtained results, the pure PLA film without nanoparticles showed no effect on the growth of the two tested bacteria. After 24 h storage time, the concentration of the two test bacteria was increased to 8.94 (*Escherichia coli*) and 9.12 log_10_CFU/mL (*Listeria monocytogenes*), respectively. On the contrary, films containing a certain percent of NPs have a significant (*p* < 0.05) effect against *E. coli* and *Listeria* bacteria at 24 h, compared with pure films. At the end of the storage time, the *E. coli* value decreased to 4.35 (PLA/Ti1%), 3.45 (PLA/Ti5%), 3.93 (PLA/Ti1%/Ag), 3.06 (PLA/Ti5%/Ag), and the *Listeria* bacteria value decreased to 4.15 (PLA/Ti1%), 3.67 (PLA/Ti5%), 3.88 (PLA/Ti1%/Ag) and 3.42 (PLA/Ti5%/Ag). Moreover, the antimicrobial ability of the film increased with the increase of the amount of nanoparticles in the PLA film. It has been reported by Díez-Pascual that the minimum TiO_2_ content required for effective microbial growth inhibition was 3.0 wt % [48]. In addition, 0.5 wt % nano-Ag added into the PLA/Ti film contributed to a certain decrease in the microbial counts. The nano-TiO_2_ could kill microbes because its particle size is very small, and it would generated many electron–hole pairs, which induced redox reactions on those microorganisms [49]. The antimicrobial mechanism of nano-Ag is that the nano-Ag particle might accumulate in the bacterial cytoplasmic membrane, and the membrane permeability of the bacteria increased significantly, which led to cell death [37,50].

### 3.9. Migration Test

One of the necessary factors to assess the applicability of the PLA nanocomposite film is the release behavior of the NPs in the application process [51]. The migration of NPs from films to 95% (*v*/*v*) aqueous ethanol and 3% (*w*/*v*) aqueous acid food simulants solution was considered as simulants of the release of NPs to acidic and alcohol-containing food.

**Figure 8:**
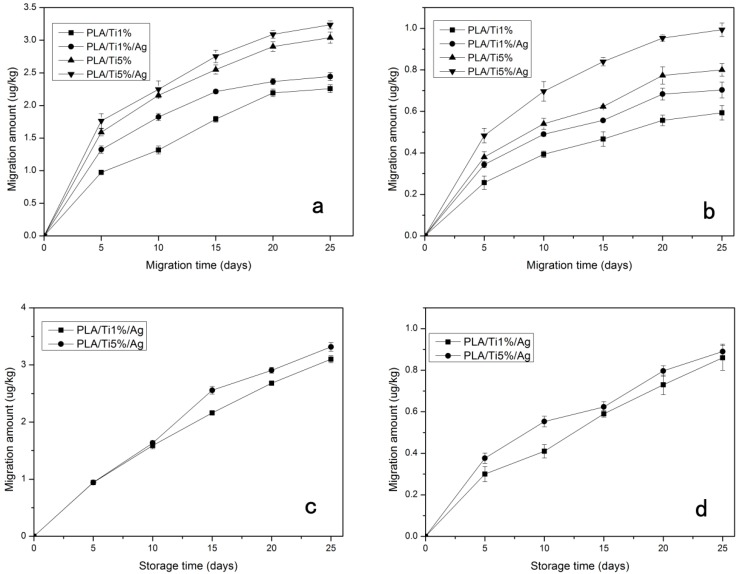
Migration of Ti from PLA/TiO_2_ film into 3% (*w*/*v*) aqueous acetic acid (**a**) and 50% (*v*/*v*) aqueous ethanol (**b**); Migration of Ag from PLA/nano-TiO_2_/nano-Ag film into 3% (*w*/*v*) aqueous acetic acid (**c**) and 50% (*v*/*v*) aqueous ethanol (**d**).

The migration amounts of Ti and Ag NPs from nano-blend film into 3% (*w*/*v*) aqueous acetic acid and 50% (*v/v*) aqueous ethanol are shown in Figure 8. The migration test results showed that as the migration time elapsed, the amount of the migrated NPs increased gradually until it reached equilibrium. Comparing the two food simulants, the nano-blend composite films differed significantly (*p* < 0.05) in the extent of migration of the nanoparticles. For Ti nanoparticles, the maximum migration ratios for 3% (*w*/*v*) aqueous acetic acid were 2.19, 2.36, 3.12 and 3.5 ug/kg for PLA/Ti1%, PLA/Ti1%/Ag, PLA/Ti5% and PLA/Ti5%/Ag, respectively. For 50% (*v*/*v*) aqueous ethanol, the maximum migration ratio amounts were 0.593, 0.72, 0.80 and0.99 ug/kg. For the 50% (*v*/*v*) aqueous ethanol, the 3% (*w*/*v*) aqueous acetic acid shows a higher amount of Ti migration. This result can be explained by simple dissolution experiments, which show that an acidic solution could more easily dissolve Ti or TiO_2_, compared to an organic solution [24]. Moreover, Ti nanoparticles migrated from all the film samples with a quick initial release rate within the first 5 days of storage. This result indicated that the initial release Ti ion came from the nanoparticle within the film surface layer or cut edges of the film, and the subsequent release of Ti ions may from the interior of the nano-blend films [15].

Ag nanoparticles showed a similar migration trend as the Ti ones. However, the release of Ag nanoparticles was almost a linear function during storage time. Since the particle size of nano-Ag is about 10 nm, which is significant lower than the particle size of nano-TiO_2_ (about 100 nm), the nano-Ag has a higher surface-to-volume ratio. Thus, the migration radio of the Ag nanoparticles was obviously higher than the Ti [52]. A similar result has been report by Song et al., who studied the migration mechanism of nano-Ag- polyethylene composite films [53]. (EU) No.10/2011 stipulates that the total content of authorized nanomaterial substances in barrier functional materials in a food product shall not exceed 1 mg·kg^−1^, thus, the NP releases of the nano-blend films were within the standard limits [54].

## 4. Conclusions

In this study, we have investigated the effect of nano-TiO_2_ and nano-Ag particles on the water vapor permeability (WVP), mechanical properties, optical properties, microstructure, thermal properties and antimicrobial properties of PLA films. We found that with the addition of the NPs in the PLA, the nano-blends film had a lower WVP and a poorer transparency, compared to the pure PLA. The introduction of NPs into the PLA film decreased the TS and EM and increased the elongation of break (ε). SEM analysis showed the cross-section of the nano-blend films became rougher. The DSC curves verified that the NPs had no effect on the T_g_ and the T_m_, but had an enhancing effect on the T_c_ and *X_c_*. With the addition of nanoparticles, TGA results showed a significant improvement in the thermal stability. PLA films containing NPs inhibited both *E. coli* and *Listeria monocytogenes* because of the bacterial inhibition ability of the NPs. Finally, we proved that the release of NPs of the nano-blend films was within the standard limits. Taken together, our results indicate that PLA film with nanoparticle fillers could be used as antimicrobial nano-blend food packaging films without food safety risks.

## Figures and Tables

**Table 1:** The mechanical properties of pure PLA and PLA nano-blend films.

Sample	Elastic Modulus (MPa)	Elongation of Break (%)	Tensile Strength (MPa)
PLA/Ti1%	3381.82 ± 26.57 ^b^	42.54 ± 2.38 ^a^^b^	11.43 ± 1.51 ^b^
PLA/Ti5%	3754.56 ± 132.15 ^c^	46.94 ± 3.64 ^a^^b^	5.73 ± 1.17 ^a^
PLA/Ti1%/Ag	3375.24 ± 131.50 ^b^	49.57 ± 3.84 ^b^	11.66 ± 3.17 ^b^
PLA/Ti5%/Ag	3554.96 ± 64.43 ^ab^	60.05 ± 7.05 ^c^	9.69 ± 3.58 ^ab^
PLA	3027.53 ± 176.41 ^a^	40.36 ± 1.74 ^a^	15.16 ± 8.89 ^c^

Values followed by different superscript letters (a–c) in the same column were significantly different (*p* < 0.05), where a is the lowest value.

**Table 2:** The color properties and opacity of pure PLA and PLA nano-blend films.

Sample	L*	a*	b*	Opacity
PLA/Ti1%	62.49 ± 0.40 ^d^	−1.19 ± 0.39 ^a^	−4.42 ± 0.41 ^b^	3.43 ± 0.05 ^b^
PLA/Ti5%	79.64 ± 0.27 ^e^	−1.00 ± 0.11 ^b^	−5.39 ± 0.26 ^a^	6.58 ± 0.08 ^d^
PLA/Ti1%/Ag	55.52 ± 0.40 ^b^	−0.20 ± 0.17 ^c^	−2.41 ± 0.29 ^d^	4.57 ± 0.04 ^c^
PLA/Ti5%/Ag	61.55 ± 0.29 ^c^	−0.76 ± 0.07 ^b^	−3.37 ± 0.10 ^c^	7.31 ± 0.04 ^e^
PLA	52.53 ± 0.29 ^a^	−1.46 ± 0.06 ^a^	−0.73 ± 0.12 ^e^	1.60 ± 0.03 ^a^

Values followed by different superscript letters (a–e) in the same column were significantly different (*p* < 0.05), where a is the lowest value.

**Table 3:** Thermal Characteristics of pure PLA and PLA nano-blend films.

Sample	T_g_ (°C)	T_c_ (°C)	T_m_ (°C)	*X_c_* (%)
PLA/Ti1%	58.4	110.9	160.5	16.5
PLA/Ti5%	58.6	113.5	160.6	19.9
PLA/Ti1%/Ag	58.0	109.3	160.0	17.9
PLA/Ti5%/Ag	58.4	110.7	160.8	18.2
PLA	58.1	125.8	160.2	11.6
